# Association of *GBA* genotype with motor and cognitive decline in Chinese Parkinson’s disease patients

**DOI:** 10.3389/fnagi.2023.1091919

**Published:** 2023-02-10

**Authors:** Jingru Ren, Gaiyan Zhou, Yajie Wang, Ronggui Zhang, Zhiying Guo, Hao Zhou, Huifen Zheng, Yu Sun, Changyan Ma, Ming Lu, Weiguo Liu

**Affiliations:** ^1^Department of Neurology, The Affiliated Brain Hospital of Nanjing Medical University, Nanjing, China; ^2^Department of Neurology, Geriatric Hospital of Nanjing Medical University, Nanjing, China; ^3^International Laboratory for Children’s Medical Imaging Research, School of Biological Sciences and Medical Engineering, Southeast University, Nanjing, China; ^4^Department of Medical Genetics, Nanjing Medical University, Nanjing, China; ^5^Jiangsu Key Laboratory of Neurodegeneration, Department of Pharmacology, Nanjing Medical University, Nanjing, China

**Keywords:** cognitive decline, GBA, longitudinal, motor impairment, Parkinson’s disease

## Abstract

**Objective:**

Variants in the glucocerebrosidase (*GBA*) gene are the most common and significant risk factor for Parkinson’s disease (PD). However, the impact of *GBA* variants on PD disease progression in the Chinese population remains unclear. This study aimed to explore the significance of *GBA* status on motor and cognitive impairment in a longitudinal cohort of Chinese patients with PD.

**Methods:**

The entire *GBA* gene was screened by long-range polymerase chain reaction (LR-PCR) and next generation sequencing (NGS). A total of 43 *GBA*-related PD (*GBA*-PD) and 246 non-*GBA*-mutated PD (NM-PD) patients with complete clinical data at baseline and at least one follow-up were recruited for this study. The associations of *GBA* genotype with rate of motor and cognitive decline, as measured by Unified PD Rating Scale (UPDRS) motor and Montreal Cognitive Assessment (MoCA), were assessed by linear mixed-effect models.

**Results:**

The estimated (standard error, SE) UPDRS motor [2.25 (0.38) points/year] and MoCA [−0.53 (0.11) points/year] progression rates in the *GBA*-PD group were significantly faster than those in the NM-PD group [1.35 (0.19); −0.29 (0.04) points/year; respectively]. In addition, the *GBA*-PD group showed significantly faster estimated (SE) bradykinesia [1.04 (0.18) points/year], axial impairment [0.38 (0.07) points/year], and visuospatial/executive [−0.15 (0.03) points/year] progression rates than the NM-PD group [0.62 (0.10); 0.17 (0.04); −0.07 (0.01) points/year; respectively].

**Conclusion:**

*GBA*-PD is associated with faster motor and cognitive decline, specifically greater disability in terms of bradykinesia, axial impairment, and visuospatial/executive function. Better understanding of *GBA*-PD progression may help predict prognosis and improve clinical trial design.

## Introduction

Parkinson’s disease (PD) is a complex chronic neurodegenerative condition (Bloem et al., 2021). Due to insufficient understanding of its pathogenesis, no disease-modifying therapies are currently available (Lang and Espay, 2018). With the relentless progression of neurodegeneration in PD, motor and cognitive impairments greatly affect patients’ quality of life and cause a huge socioeconomic burden (Feigin et al., 2019). Given that motor and cognitive deterioration rates vary considerably among PD patients (Armstrong and Okun, 2020), identifying the genes that modulate disease progression is potentially important for improving trial design—especially in neuroprotective therapies—and developing personalized treatment approaches.

One of the most effective ways to identify genetic modifiers of PD progression is to examine established PD susceptibility genes (Bandres-Ciga et al., 2020). Heterozygous variants in the glucocerebrosidase (*GBA*; OMIM 606463) gene, which encodes the lysosomal enzyme β-glucocerebrosidase (GCase), have been identified as the most common and significant genetic risk factor for PD worldwide (Sidransky et al., 2009). The frequency of *GBA* variants is ethnically heterogeneous, estimated to be 10–31% in Ashkenazi Jewish (AJ), 3–12% in non-AJ North Americans, and 2–11% in Chinese populations (Neumann et al., 2009; Mao et al., 2010; Gan-Or et al., 2015; Li et al., 2020; Ren et al., 2022). Notably, a highly homologous pseudogene (*GBAP1*), located 16 kb downstream, shares 96% sequence identity with the functional *GBA* gene, making *GBA* sequencing more challenging (Horowitz et al., 1989; Hruska et al., 2008).

Clinically, *GBA*-related PD (*GBA*-PD) is characterized by an earlier age of onset, more severe motor and non-motor symptoms (specifically cognitive decline, rapid eye movement sleep disorder, depression, and autonomic dysfunction), and lower survival rates compared with non-*GBA*-mutated PD (NM-PD) (Brockmann et al., 2015; Malek et al., 2018; Stoker et al., 2020; Zhao et al., 2020). Importantly, *GBA* variant severity underlies the phenotypic spectrum of PD (Petrucci et al., 2020; Ren et al., 2022). Several studies have shown that *GBA* status is an independent risk factor for cognitive decline in PD and that *GBA* genotype severity is linked to the rate of cognitive impairment progression (Cilia et al., 2016; Liu et al., 2016; Lunde et al., 2018; Stoker et al., 2020; Szwedo et al., 2022). However, the distinct pattern of progression in cognitive impairment in *GBA*-PD patients remains unclear. Although PD is predominantly a movement disorder, few studies have examined the impact of *GBA* status on progression of motor decline (Davis M. Y. et al., 2016; Ortega et al., 2021). In a recent study of Northern European PD patients, *GBA* variants were associated with more aggressive motor impairment (Maple-Grødem et al., 2021).

To the best of our knowledge, the influence of *GBA* variants on motor and cognitive impairment has not been previously investigated in the Chinese PD population. To address this gap, we studied a longitudinal cohort of Chinese PD patients to comprehensively explore the role of *GBA* status in the progression of motor and cognitive decline, especially patterns of motor and cognitive progression.

## Materials and methods

### Participants

This prospective comprehensive study of a large cohort of Chinese patients with *GBA*-PD or NM-PD was conducted at the Department of Neurology of the Affiliated Brain Hospital of Nanjing Medical University from 2012 to 2022. The inclusion criteria were: (1) a diagnosis of PD by a movement disorder specialist according to the United Kingdom PD Society Brain Bank clinical diagnostic criteria (Gibb and Lees, 1988), regardless of the presence of a family history of PD; and (2) received genetic testing for *GBA* variants. The exclusion criteria were: (1) diagnosed with atypical or secondary parkinsonism disorders; (2) a history of serious chronic diseases, such as kidney or heart failure; and (3) brain magnetic resonance imaging (MRI) scans revealing clinically significant lesions. Patients who met these criteria were invited to participate in in-depth annual follow-up at our institution if available.

A total of 737 unrelated PD patients were recruited from January 2012 to June 2020. Of these 79 (10.7%) were confirmed to have *GBA* variants and the remaining 658 (89.3%) had no *GBA* variants. Among the 79 patients with *GBA* variants, 43 were followed up at least once with complete clinical data at baseline (*GBA*-PD group). Of the 658 patients without *GBA* variants, 246 were followed up at least once with complete clinical data at baseline (NM-PD group) (Supplementary Figure 1).

This study was approved by the Medical Ethics Committee of the Affiliated Brain Hospital of Nanjing Medical University. All participants provided written informed consent prior to entering the study.

### Clinical evaluation

Standardized procedures to collect detailed demographic and clinical data were conducted through face-to-face interviews with all PD patients. The levodopa equivalent daily dose (LEDD) was calculated in accordance with an established method (Tomlinson et al., 2010). The Unified PD Rating Scale (UPDRS) part II, part III, and modified Hoehn–Yahr (H–Y) stage were used to assess activities of daily living (ADL), motor impairment, and disease severity, respectively. The severity of specific motor characteristics in PD patients was determined by summing the following relevant UPDRS III items: tremor (items 20 and 21), rigidity (item 22), bradykinesia (items 23–26 and 31), and axial impairment (item 27–30). Cognitive ability was measured with the Mini-Mental State Examination (MMSE) and Montreal Cognitive Assessment (MoCA). The MoCA comprises 30 items divided into 7 domains, namely, visuospatial/executive (5 points), naming (3 points), attention (6 points), language (3 points), abstraction (2 points), delayed recall (5 points), and orientation (6 points). Total MoCA scores were adjusted for education by adding one point for individuals with ≤12 years of education (Nasreddine et al., 2005).

### Analysis of *GBA* genetic variants

The entire *GBA* gene was screened using the long-range polymerase chain reaction (LR-PCR) protocol to avoid nearby *GBAP1* contamination followed by next generation sequencing (NGS), as previously described (Ren et al., 2022). All identified variants were confirmed by repeating amplification and resequencing. We adopted the traditional nomenclature for *GBA* alleles, referring to the processed protein and excluding the 39-residue signal peptide. Similar to previous studies (Liu et al., 2016; Petrucci et al., 2020; Ren et al., 2022), *GBA* variants were divided into five types: mild [causing Gaucher disease (GD) type 1], severe (causing GD type 2 or 3), risk (associated with risk factors of PD, but meaningless for GD), complex (≥2 variants, with no confirmation of *cis* distribution), and variants of unknown significance (VUS). Pathogenicity predictions of the identified non-synonymous *GBA* variants were assessed with 22 computational methods, including 9 function-prediction methods (FATHMM, fitCons, LRT, MutationAssessor, MutationTaster, PolyPhen2-HVAR, PROVEAN, SIFT, and VEST3), 4 conservation methods (GERP++, phastCons, phyloP, and SiPhy), and 9 ensemble methods (CADD, DANN, Eigen, FATHMM-MKL, GenoCanyon, M-CAP, MetaLR, MetaSVM, and REVEL) (Supplementary Table 1). Scores for the 22 methods were downloaded directly from the dbNSFP database v3. The cutoffs used to distinguish deleterious non-synonymous variants were adopted from a previous study (Li et al., 2018).

### Statistical analysis

Statistical analyses were performed using IBM SPSS Statistics version 27.0 or Stata version 17.0. Baseline between-group differences were compared using linear regression analysis for continuous variables and logistic regression analysis for binary variables, adjusting for the confounders listed in the Table 1. Linear mixed-effects models were used to examine the relationship between *GBA* genotype and longitudinal motor and cognitive measures assessed as repeat UPDRS motor and MoCA scores. As linear mixed-effects models can accommodate unbalanced data due to missing data points, dropout, unequal visit intervals, and staggered first visit times and account for the correlation between repeated measures across individuals, they are often used to analyze the trajectory of longitudinal markers. Disease duration was used as the time scale considering its correlation with motor and cognitive function. A participant-specific random effect was used to explain repeated-measures correlations within the same participant. Time, *GBA* genotype, and their interaction were included as fixed effects. Motor and cognitive progression was modeled adjusting for sex, age at baseline, and LEDD or education. All models had random intercepts of the participants’ IDs as well as a random slope of time. Predictive margins of motor and cognitive decline were plotted using margin and marginsplot in Stata. Findings with two-tailed *P*-values < 0.05 were considered statistically significant.

**TABLE 1:** Baseline demographic and clinical features of *GBA*-related PD (*GBA*-PD) and non-*GBA* mutated PD (NM-PD) patients.

Variable	*GBA*-PD (*n* = 43)	NM-PD (*n* = 246)	*p*-value^a^
Age at baseline (years)	56.9 ± 9.6	59.8 ± 8.3	**0.039** ^b^
Age at onset (years)	53.2 ± 10.5	56.7 ± 8.6	**0.040** ^b^
Disease duration (years)	3.7 ± 3.3	3.2 ± 2.9	0.180^c^
Male [*n* (%)]	20 (46.5)	137 (55.7)	0.194^d^
Formal education (years)	9.7 ± 4.0	10.4 ± 4.1	0.404
LEDD (mg/day)	349.8 ± 291.8	260.2 ± 268.4	0.083
H–Y stage	2.0 ± 0.9	1.7 ± 0.7	**0.049** ^e^
UPDRS ADL score	10.6 ± 6.8	8.8 ± 4.7	0.076^e^
UPDRS motor score	26.3 ± 13.9	20.9 ± 10.3	**0.003** ^e^
Tremor	3.6 ± 2.6	3.3 ± 2.8	0.513^e^
Rigidity	6.5 ± 5.2	4.2 ± 3.4	**<0.001** ^e^
Bradykinesia	11.0 ± 7.3	8.8 ± 5.3	**0.017** ^e^
Axial impairment	3.4 ± 2.3	3.1 ± 1.9	0.556^e^
MMSE score	27.1 ± 3.6	27.4 ± 3.1	0.744^f^
MoCA score	23.7 ± 4.6	23.8 ± 4.5	0.985^f^
Visuospatial/Executive	2.9 ± 1.6	3.3 ± 1.5	0.155^f^
Naming	2.7 ± 0.6	2.7 ± 0.6	0.533^f^
Attention	5.3 ± 1.0	5.3 ± 1.1	0.605^f^
Language	2.4 ± 0.9	2.4 ± 0.8	0.703^f^
Abstraction	1.4 ± 0.8	1.4 ± 0.8	0.835^f^
Recall	2.3 ± 1.6	2.2 ± 1.6	0.919^f^
Orientation	5.7 ± 0.5	5.8 ± 0.7	0.804^f^

Data are reported as mean ± SD or n (%). *P*-values were calculated using linear regression or logistic regression.

ADL, activities of daily living; *GBA*-PD, *GBA*-related PD; H–Y, Hoehn–Yahr; LEDD, levodopa equivalent daily dose; MMSE, mini-mental state examination; MoCA, Montreal Cognitive Assessment; NM-PD, non-*GBA*-mutated PD; PD, Parkinson’s disease; UPDRS, Unified Parkinson’s Disease Rating Scale.

^a^Adjusted for age, sex, and disease duration (unless otherwise indicated).

^b^Adjusted for sex and disease duration.

^c^Adjusted for age and sex.

^d^Adjsuted for age and disease duration.

^e^Adjusted for age, sex, disease duration, and LEDD.

^f^Adjusted for age, sex, disease duration, and years of formal education.

Values in bold indicate statistically significant differences (*P* < 0.05).

## Results

### *GBA* variants

A total of 31 distinct variants were identified in the 43 *GBA*-PD patients (Supplementary Table 2). Of these 43 cases, the variant severity classes were 3 mild, 16 severe, 1 risk, 3 complex, and 20 unknown. The *in silico* pathogenicity predictions of the identified non-synonymous *GBA* variants are presented in Supplementary Table 1. Only D399H and W393C were predicted to be deleterious by all 22 computational methods. Notably, R163Q, a common *GBA* variant in the Chinese population, was predicted to be tolerable by 14 computational methods in the *in silico* analyses.

### Baseline profile of *GBA*-PD patients

Table 1 presents the baseline demographic and clinical profiles of the *GBA*-PD and NM-PD groups. A total of 289 patients (43 carriers and 246 non-carriers) were included in this analysis. The mean follow-up time was 2.6 years in the *GBA*-PD group and 3.5 years in the NM-PD group. Consistent with our previous report (Ren et al., 2022), PD patients with and without *GBA* mutations were similar in terms of disease duration, sex, formal education, LEDD, ADL, and cognitive impairment. Compared with NM-PD patients, *GBA*-PD patients had a younger baseline age, earlier age of onset, greater modified H–Y stages, and more severe UPDRS motor scores, adjusting for potential confounding factors. In addition, *GBA*-PD patients had more severe rigidity and bradykinesia scores than NM-PD patients. However, PD patients with and without *GBA* mutations did not differ in terms of severity of tremor or axial impairment.

### Effect of *GBA* on motor decline

**FIGURE 1:**
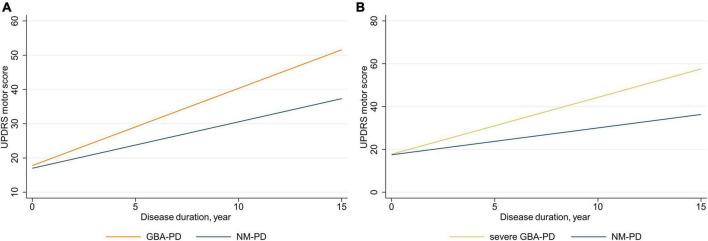
Longitudinal trajectories of mean Unified Parkinson’s Disease Rating Scale (UPDRS) motor scores. **(A)** *GBA*-PD vs. NM-PD; **(B)** severe *GBA*-PD vs. NM-PD. *GBA*-PD, *GBA*-related PD; NM-PD, non-*GBA*-mutated PD; PD, Parkinson’s disease.

Linear mixed-effects models were used to examine the association of *GBA* genotype with the rate of change in UPDRS motor scores and subscores, adjusting for sex, age at baseline, and LEDD at visit (Figure 1 and Table 2). The estimated (standard error, SE) progression rate of UPDRS motor score in the *GBA*-PD group [2.25 (0.38) points/year; *P* < 0.001] was significantly faster than that in the NM-PD group [1.35 (0.19) points/year; *P* < 0.001] [difference, 0.90 (0.37) points/year; *P* = 0.017] (Supplementary Figure 2). For *GBA*-PD, this remained significant after excluding VUS [difference, 1.64 (0.45) points/year; *P* < 0.001]. In addition, the progression rate of UPDRS motor score did not differ between the non-severe *GBA*-PD and NM-PD groups [difference, 0.73 (0.44) points/year; *P* = 0.099], whereas the estimated (SE) progression rate of UPDRS motor score in the severe *GBA*-PD group [2.65 (0.61) points/year; *P* < 0.001] was significantly faster than that in the NM-PD group [1.25 (0.18) points/year; *P* < 0.001] [difference, 1.40 (0.60) points/year; *P* = 0.020]. The magnitude of the impact on motor impairment in the severe *GBA*-PD group was greater than that in the overall *GBA*-PD group.

**TABLE 2:** Association between *GBA* carrier status and annual change in Unified Parkinson’s Disease Rating Scale (UPDRS) motor scores and subscores using linear mixed-effects models.

	*GBA*-PD (*n* = 43)^a^		*GBA*-PD without VUS (*n* = 23)^a^		Severe *GBA*-PD (*n* = 16)^a^	
Characteristic^b^	β (95% CI)	*p*-value	β (95% CI)	*p*-value	β (95% CI)	*p*-value
**UPDRS motor**						
Main effect	0.81 (−3.47, 5.09)	0.711	−2.21 (−7.62, 3.21)	0.424	0.30 (−5.82, 6.42)	0.923
Interaction with time	**0.90 (0.16, 1.63)**	**0.017**	**1.64 (0.76, 2.52)**	**<0.001**	**1.40 (0.22, 2.57)**	**0.020**
**Tremor**						
Main effect	−0.01 (−1.10, 1.07)	0.984	−0.35 (−1.76, 1.05)	0.621	−0.07 (−1.68, 1.54)	0.934
Interaction with time	0.10 (−0.07, 0.27)	0.241	0.15 (−0.06, 0.37)	0.167	0.06 (−0.23, 0.35)	0.700
**Rigidity**						
Main effect	1.43 (−0.09, 2.94)	0.066	−0.1 (−2.03, 1.82)	0.915	0.28 (−1.92, 2.49)	0.801
Interaction with time	0.08 (−0.20, 0.35)	0.581	0.31 (−0.04, 0.65)	0.080	0.30 (−0.16, 0.75)	0.201
**Bradykinesia**						
Main effect	0.05 (−2.26, 2.35)	0.969	−0.57 (−3.48, 2.34)	0.700	0.89 (−2.39, 4.18)	0.594
Interaction with time	**0.41 (0.05, 0.77)**	**0.024**	**0.75 (0.33, 1.16)**	**<0.001**	**0.62 (0.05, 1.18)**	**0.033**
**Axial impairment**						
Main effect	−0.38 (−1.14, 0.39)	0.336	−0.53 (−1.49, 0.43)	0.282	−0.52 (−1.64, 0.59)	0.357
Interaction with time	**0.20 (0.06, 0.35)**	**0.005**	**0.22 (0.04, 0.4)**	**0.018**	**0.26 (0.02, 0.51)**	**0.033**

CI, confidence interval; *GBA*-PD, *GBA*-related PD; NM-PD, non-GBA-mutated PD; PD, Parkinson’s disease; VUS, variants of unknown significance.

^a^*GBA*-PD compared to NM-PD: UPDRS motor scores and subscores were available for 246 NM-PD and 43 *GBA*-PD patients.

^b^Models adjusted for sex, age at baseline, and levodopa equivalent daily dose (LEDD) at visit. The main effect indicates the effect of *GBA* carrier status on the intercept, while the interaction with time indicates the effect of *GBA* carrier status on the slope (annual change in UPDRS motor scores and subscores). Values in bold are statistically significant (*P* < 0.05).

Differences in motor decline were driven primarily by changes in bradykinesia and axial impairment scores. The estimated (SE) progression rate of bradykinesia score in the *GBA*-PD group [1.04 (0.18) points/year; *P* < 0.001] was significantly faster than that in the NM-PD group [0.62 (0.10) points/year; *P* < 0.001] [difference, 0.41 (0.18) points/year; *P* = 0.024]. Furthermore, the estimated (SE) progression rate of bradykinesia score in the severe *GBA*-PD group [1.17 (0.30) points/year; *P* < 0.001] was significantly faster than that in the NM-PD group [0.56 (0.09) points/year; *P* < 0.001] [difference, 0.62 (0.29) points/year; *P* = 0.033]. Similarly, the estimated (SE) progression rate of axial impairment score in the *GBA*-PD group [0.38 (0.07) points/year; *P* < 0.001] was significantly faster than that in the NM-PD group [0.17 (0.04) points/year; *P* < 0.001; difference, 0.20 (0.07) points/year; *P* = 0.005], and the estimated (SE) progression rate of axial impairment score in the severe *GBA*-PD group [0.42 (0.13) points/year; *P* = 0.001] was significantly faster than that in the NM-PD group [0.16 (0.04) points/year; *P* < 0.001] [difference, 0.26 (0.12) points/year; *P* = 0.033].

### Effect of *GBA* on cognitive decline

**FIGURE 2:**
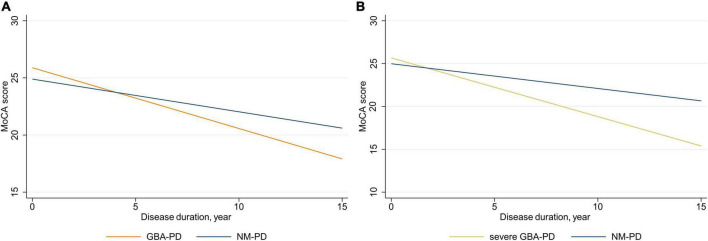
Longitudinal trajectories of mean Montreal Cognitive Assessment (MoCA) scores. **(A)** *GBA*-PD vs. NM-PD; **(B)** severe *GBA*-PD vs. NM-PD. *GBA*-PD, *GBA*-related PD; NM-PD, non-*GBA*-mutated PD; PD, Parkinson’s disease.

Next, we used linear mixed-effects models to explore the association of *GBA* genotype with the rate of annual decline in MoCA scores and subscores adjusted for sex, age at baseline, and education (Figure 2 and Table 3). The estimated (SE) progression rate of MoCA score in the *GBA*-PD group [−0.53 (0.11) points/year; *P* < 0.001] was significantly faster than that in the NM-PD group [−0.29 (0.04) points/year; *P* < 0.001] [difference, −0.25 (0.12) points/year; *P* = 0.035]. For *GBA*-PD, this remained significant after excluding VUS [difference, −0.40 (0.15) points/year; *P* = 0.009]. Furthermore, the progression rate of MoCA motor score did not differ between the non-severe *GBA*-PD and NM-PD groups [difference, −0.20 (0.14) points/year; *P* = 0.147], whereas the estimated (SE) progression rate of MoCA score in the severe *GBA*-PD group [−0.68 (0.19) points/year; *P* < 0.001] was significantly faster than that in the NM-PD group [−0.29 (0.04) points/year; *P* < 0.001] [difference, −0.40 (0.20) points/year; *P* = 0.046]. The magnitude of the impact on cognitive impairment in the severe *GBA*-PD group was greater than that in the entire *GBA*-PD group.

**TABLE 3:** Association between *GBA* carrier status and annual change in Montreal Cognitive Assessment (MoCA) scores and subscores using linear mixed-effects models.

	*GBA*-PD (*n* = 43)^a^		*GBA*-PD without VUS (*n* = 23)^a^		Severe *GBA*-PD (*n* = 16)^a^	
Characteristic^b^	β (95% CI)	*p*-value	β (95% CI)	*p*-value	β (95% CI)	*p*-value
**MoCA**						
Main effect	0.99 (−0.61, 2.6)	0.225	1.38 (−0.72, 3.49)	0.198	0.67 (−1.71, 3.06)	0.579
Interaction with time	**−0.25 (−0.47, −0.02)**	**0.035**	**−0.4 (−0.69, −0.1)**	**0.009**	**−0.4 (−0.78, −0.01)**	**0.046**
**Visuospatial/executive**						
Main effect	−0.07 (−0.56, 0.43)	0.790	0.31 (−0.33, 0.95)	0.339	0.28 (−0.43, 1.00)	0.435
Interaction with time	**−0.08 (−0.15, 0.00)**	**0.040**	**−0.14 (−0.23, −0.05)**	**0.003**	**−0.15 (−0.27, −0.03)**	**0.016**
**Naming**						
Main effect	0.10 (−0.12, 0.33)	0.370	0.04 (−0.24, 0.32)	0.784	0.05 (−0.27, 0.37)	0.754
Interaction with time	−0.03 (−0.06, 0.01)	0.134	−0.02 (−0.06, 0.02)	0.296	−0.02 (−0.08, 0.03)	0.375
**Attention**						
Main effect	0.03 (−0.41, 0.47)	0.893	0.05 (−0.51, 0.61)	0.869	0.24 (−0.40, 0.88)	0.458
Interaction with time	−0.03 (−0.09, 0.04)	0.430	−0.03 (−0.11, 0.05)	0.482	−0.08 (−0.19, 0.03)	0.146
**Language**						
Main effect	0.24 (−0.05, 0.53)	0.102	0.05 (−0.32, 0.42)	0.797	−0.07 (−0.5, 0.36)	0.753
Interaction with time	−0.03 (−0.08, 0.01)	0.146	−0.02 (−0.07, 0.04)	0.603	−0.02 (−0.1, 0.06)	0.621
**Abstraction**						
Main effect	0.20 (−0.05, 0.44)	0.121	0.22 (−0.1, 0.54)	0.185	0.10 (−0.27, 0.46)	0.605
Interaction with time	−0.02 (−0.06, 0.01)	0.200	−0.04 (−0.09, 0)	0.063	−0.04 (−0.10, 0.02)	0.182
**Recall**						
Main effect	0.35 (−0.24, 0.94)	0.248	0.33 (−0.43, 1.1)	0.398	0.23 (−0.65, 1.1)	0.611
Interaction with time	−0.06 (−0.15, 0.02)	0.159	−0.09 (−0.2, 0.02)	0.107	−0.11 (−0.26, 0.04)	0.146
**Orientation**						
Main effect	0.03 (−0.29, 0.34)	0.871	0.01 (−0.41, 0.43)	0.958	0.06 (−0.42, 0.54)	0.794
Interaction with time	−0.00 (−0.05, 0.05)	0.972	−0.01 (−0.07, 0.06)	0.866	−0.02 (−0.11, 0.07)	0.643

CI, confidence interval; *GBA*-PD, GBA-related PD; NM-PD, non-GBA-mutated PD; PD, Parkinson’s disease; VUS, variants of unknown significance.

^a^*GBA*-PD compared to NM-PD: MoCA scores and subscores were available for 222 NM-PD and 43 *GBA*-PD patients.

^b^Models adjusted for sex, age at baseline and education. The main effect indicates the effect of *GBA* carrier status on the intercept, while the interaction with time indicates the effect of GBA carrier status on the slope (annual change in MoCA scores and subscores). Values in bold are statistically significant (*P* < 0.05).

Differences in cognitive decline were driven primarily by changes in visuospatial/executive score. The estimated (SE) progression rate of visuospatial/executive score in the *GBA*-PD group [−0.15 (0.03) points/year; *P* < 0.001] was significantly faster than that in the NM-PD group [−0.07 (0.01) points/year; *P* < 0.001] [difference, −0.08 (0.04) points/year; *P* = 0.040]. Further, the estimated (SE) progression rate of visuospatial/executive score in the severe *GBA*-PD group [−0.22 (0.06) points/year; *P* < 0.001] was significantly faster than that in the NM-PD group [−0.07 (0.01) points/year; *P* < 0.001] [difference, −0.15 (0.06) points/year; *P* = 0.016].

## Discussion

To our knowledge, this is the first paper to systematically assess the association of *GBA* genotype with long-term motor and cognitive decline in a longitudinal Chinese PD cohort. The results showed that both motor and cognitive impairment progressed more rapidly in the *GBA*-PD patients, especially the severe subgroup, compared to the NM-PD patients. Furthermore, examination of changes in specific motor and cognitive domains revealed that between-group differences in progression were primarily attributable to bradykinesia, axial impairment, and visuospatial/executive functioning.

UPDRS motor score, which is an operational standard for assessing symptom severity, has the advantage of good intra- and inter-rater reliability, making it one of the most used primary outcomes in observational studies and clinical trials. However, surprisingly few studies have used UPDRS motor score to evaluate progression of motor impairment. In our study, *GBA*-PD patients showed faster motor decline, as measured by rate of change in UPDRS motor score, consistent with recent longitudinal findings (Davis M. Y. et al., 2016; Maple-Grødem et al., 2021; Ortega et al., 2021). Furthermore, we found that severe *GBA*-PD patients experienced more rapid motor progression than overall *GBA*-PD patients, compared to NM-PD patients, implying that motor impairment progression is associated with *GBA* variant severity. In contrast to our findings, (Davis A. A. et al., 2016) found no significant association between either variant (N370S or K26R) and annual change in UPDRS motor score in the “on” or “off” state in PD patients from two cohorts. These inconsistent findings could relate to the fact that the *GBA*-PD group in our study included all *GBA* variants, rather than analysis of individual *GBA* variants. Considering the low frequency of individual *GBA* variants, greater collaboration is required to study individual *GBA* variants. In the largest genome-wide association study to date involving 12 cohorts, T369M was associated with faster progression of motor impairment severity to H–Y stage 3 (Iwaki et al., 2019). Alternatively, as done in this study, screening the entire *GBA* gene and classifying *GBA* severity, in the absence of *GBAP1* interference, could clarify whether *GBA* status affects motor decline progression in PD patients.

Numerous studies have reported an association between *GBA* variants and more rapid progression of cognitive symptoms, as assessed using the MMSE, in PD patients (Cilia et al., 2016; Liu et al., 2016; Lunde et al., 2018; Stoker et al., 2020; Szwedo et al., 2022). However, there is a paucity of longitudinal studies using the MoCA to evaluate cognitive features in *GBA*-PD patients (Ortega et al., 2021). It is well known that the MoCA is more sensitive than the MMSE for detecting mild, domain-specific changes in global cognitive performance in PD (Hoops et al., 2009; Skorvanek et al., 2018). Thus, our finding that *GBA*-PD patients experienced faster cognitive decline, as represented by annual change in MoCA, provides further evidence that *GBA* is a major risk factor for cognitive impairment. We also observed that severe *GBA*-PD patients had faster cognitive progression than entire *GBA*-PD patients, relative to NM-PD patients. Although previous studies have used different classification methods for *GBA* variants, it has been consistently demonstrated that the progression of cognitive impairment increases with *GBA* variant severity (Cilia et al., 2016; Liu et al., 2016; Lunde et al., 2018; Szwedo et al., 2022). Several studies have reported no link between *GBA* risk variants and cognitive impairment, which may be attributed to the small sample sizes or combining E326K and T369M variants with synonymous or intronic variants, which dilutes the effect of risk variants (Winder-Rhodes et al., 2013; Jesús et al., 2016). Recent longitudinal studies have demonstrated that cognitive impairment progresses more rapidly in risk *GBA*-PD patients (Davis M. Y. et al., 2016; Lunde et al., 2018; Straniero et al., 2020; Szwedo et al., 2022). Therefore, both severe and risk *GBA* variants appear to modify cognitive progression in PD and could help explain clinical heterogeneity among *GBA*-PD patients.

Since *GBA*-PD is associated with faster progression of motor decline and global cognitive impairment, we further investigated whether and to what extent *GBA* status was related to changes in specific motor features and cognitive domains. We found that progression rates for subscores related to bradykinesia, axial impairment, and visuospatial/executive function differed significantly between groups. Regarding specific motor symptoms, a similar study reported that *GBA* variants were significantly associated with worsening of bradykinesia and axial impairment (Maple-Grødem et al., 2021), which is comparable to our results. It is well known that cognitive impairment is a clinical signature of *GBA*-PD. Bradykinesia and rigidity impairment are recognized as independent risk factors for cognitive impairment in PD (Aarsland et al., 2021), which explains why *GBA*-PD patients show rapid progression in these areas. Regarding specific cognitive domains, our results are consistent with those of a recent 10-year longitudinal study of 10 *GBA*-PD and 20 NM-PD patients reporting that *GBA*-PD patients had greater deterioration in visuospatial function compared with NM-PD patients (Leocadi et al., 2022). In a previous multicenter cross-sectional study, *GBA*-PD patients showed a unique cognitive pattern of significant impairment of working memory/executive function and visuospatial abilities (Mata et al., 2016). Our data provide an important missing piece of the puzzle, demonstrating for the first time in a large longitudinal study that cognitive progression in *GBA*-PD is specifically manifested as visuospatial/executive function impairments.

This study has several strengths, including (1) establishing the first *GBA*-PD longitudinal cohort in a Chinese population; (2) demonstrating that the pattern of rapid motor and cognitive progression in *GBA*-PD is characterized by severely impaired bradykinesia, axial impairment, and visuospatial/executive function; and (3) screening the entire *GBA* gene without *GBAP1* interference for more accurate variant-specific analysis. However, there are also several limitations that should be considered. (1) An insufficient sample size for the genotype group precluded assessment of mild or risk *GBA* variants. Larger longitudinal studies are thus needed to determine whether motor or cognitive progression rates are affected by mild or risk *GBA* variations. (2) All PD patients in this study underwent *GBA* gene testing. However, some PD patients may have common PD-related genes, such as *LRRK2*, *PRKN*, *APOE*, and *PICALM*, which may have influenced the results. Therefore, it is necessary to exclude common PD-related genes. (3) Lastly, we used MoCA for cognitive screening and did not perform detailed neuropsychological tests of each cognitive domain, as suggested by the Movement Disorder Society (Litvan et al., 2012).

## Conclusion

This is the first longitudinal follow-up study to comprehensively assess motor and cognitive progression in Chinese *GBA*-PD patients. We found that *GBA*-PD is associated with faster motor and cognitive decline, which was specifically characterized by greater impairment in bradykinesia, axial impairment, and visuospatial/executive function. These findings have important clinical implications for understanding the role of *GBA* variants on patterns of motor and cognitive decline in the natural course of PD, which could help clinicians more accurately predict prognosis and design clinical trials for potential disease-modifying therapies.

## Data availability statement

The original contributions presented in this study are included in this article/Supplementary material, further inquiries can be directed to the corresponding author.

## Ethics statement

The studies involving human participants were reviewed and approved by the Ethics Committee of the Affiliated Brain Hospital of Nanjing Medical University. The patients/participants provided their written informed consent to participate in this study.

## Author contributions

WL organized the project and critically revised the manuscript. JR organized the project, drafted the preliminary manuscript, and performed statistical analysis. GZ, YW, and RZ collected data and performed statistical analysis. ZG, HaZ, HuZ, and YS critiqued the statistical analysis and interpreted the data. CM and ML critically revised the manuscript. All authors contributed to the article and approved the submitted manuscript.
