# Supplementary material for: Association of GBA genotype with motor and cognitive decline in Chinese Parkinson’s disease patients
Source: Front Aging Neurosci. 2023 Feb 10;15:1091919. doi: 10.3389/fnagi.2023.1091919 (PMC9950580; doi:10.3389/fnagi.2023.1091919)
Supplement: Supplementary file 1 [file Table_1.docx]

**Supplementary Materials**

**Supplementary Table**

**Supplementary Table 1.** Pathogenicity predictions of the identified non-synonymous *GBA* variants in the computational methods

**Supplementary Table 2.** *GBA* variants identified in *GBA*-PD group

**Supplementary Figure**

**Supplementary Figure 1.** The flow chart of participant screening

**Supplementary Figure 2.** Spaghetti plots of changes in UPDRS motor score across visits

**Supplementary Table 1.** Pathogenicity predictions of the identified non-synonymous *GBA* variants in the computational methods

| **Allele name** | **Function prediction methods** | | | | | | | | | **Conservation methods** | | | | **Ensemble methods** | | | | | | | | |  |
| --- | --- | --- | --- | --- | --- | --- | --- | --- | --- | --- | --- | --- | --- | --- | --- | --- | --- | --- | --- | --- | --- | --- | --- |
|  | FATHMM | fitCons | LRT | Mutation  Assessor | Mutation  Taster | Polyphen2_HVAR | PROVEAN | SIFT | VEST3 | GERP++ | phastCons | PhyloP | SiPhy | CADD | DANN | Eigen | FATHMM -MKL | GenoCanyon | M-CAP | MetaLR | MetaSVM | REVEL | |
|  | D ≤ -1.5 | D > 0.7 | D ≤ 0.001 | D > 1.9 |  | D ≥ 0.447 | D ≤ -2.5 | D ≤ 0.05 | D ≥ 0.5 | D ≥ 2 | D > 0.999 | D > 2 | D ≥ 12 | D > 20 | D ≥ 0.99 | D ≥ 0 | D > 0.5 | D > 0.999 | D > 0.025 | D > 0.5 | D > 0 | D ≥ 0.4 | |
| D380N | D (-5.56) | D (0.732) | D (0) | D (3.19) | D (1) | D (0.999) | D (-4.75) | D (0) | T (0.415) | D (3.53) | D (1) | D (7.067) | D (12.957) | D (34) | D (0.999) | D (0.652) | D (0.993) | D (1) | D (0.927) | D (0.983) | D (1.043) | D (0.942) | |
| D399H | D (-6.33) | D (0.707) | D (0) | D (3.42) | D (1) | D (1) | D (-6.11) | D (0.002) | D (0.99) | D (4.95) | D (1) | D (7.172) | D (16.05) | D (27.3) | D (0.995) | D (0.673) | D (0.996) | D (1) | D (0.924) | D (0.989) | D (1.008) | D (0.936) | |
| E326K | D (-5.77) | D (0.707) | T (0.075) | T (1.005) | D (0) | T (0.043) | T (-1.2) | T (0.784) | T (0.402) | D (3.67) | T (0.003) | T (0.321) | T (11.005) | T (17.33) | T (0.985) | T (-0.392) | T (0.154) | T (0.078) | NA | D (0.85) | D (0.675) | D (0.595) | |
| F213I | D (-5.59) | D (0.706) | D (0.001) | D (2.255) | D (0.998) | T (0.077) | D (-4.54) | D (0.002) | D (0.904) | D (3.67) | D (1) | D (6.352) | T (8.892) | D (23.6) | T (0.975) | D (0.082) | D (0.974) | D (1) | D (0.807) | D (0.912) | D (1.083) | D (0.786) | |
| G202R | D (-5.5) | D (0.706) | D (0) | D (2.3) | D (1) | T (0.421) | D (-5.08) | T (0.12) | D (0.95) | D (3.66) | D (1) | D (5.202) | T (11.069) | D (24.4) | T (0.986) | D (0.132) | D (0.945) | D (1) | D (0.594) | D (0.91) | D (1) | D (0.798) | |
| G325R | D (-5.4) | D (0.707) | D (0) | T (1.205) | D (0.741) | T (0.013) | T (-1.44) | D (0.029) | D (0.883) | T (1.7) | T (0.185) | T (1.161) | T (4.78) | T (18.92) | T (0.868) | T (-0.352) | D (0.869) | T (0.022) | D (0.147) | D (0.774) | D (0.154) | D (0.593) | |
| G377C | D (-6.6) | D (0.732) | D (0) | D (3.82) | D (1) | D (1) | D (-7.89) | D (0.001) | D (0.869) | D (3.53) | T (0.999) | D (4.311) | D (12.957) | D (34) | D (0.997) | D (0.535) | D (0.984) | D (1) | D (0.923) | D (0.99) | D (0.984) | D (0.96) | |
| L(-25)S | D (-5.7) | T (0.543) | T (0.027) | T (0.205) | T (1) | T (0) | T (-0.26) | T (0.459) | T (0.131) | T (-6.63) | T (0) | T (-3.613) | T (3.327) | T (0.001) | T (0.386) | T (-2.269) | T (0.004) | D (1) | D (0.168) | D (0.69) | D (0.266) | T (0.313) | |
| L185R | D (-6.37) | D (0.706) | D (0.001) | D (2.8) | D (0.999) | D (0.985) | D (-3.9) | D (0.001) | D (0.874) | D (3.66) | D (1) | D (8.099) | T (8.876) | D (24.1) | D (0.996) | D (0.37) | D (0.978) | D (1) | D (0.899) | D (0.981) | D (1.069) | D (0.891) | |
| L324V | D (-6.26) | D (0.707) | D (0) | D (1.925) | D (0.999) | D (0.913) | T (-2.38) | T (0.09) | D (0.715) | T (1.18) | T (0.904) | T (1.253) | T (5.31) | D (21.4) | D (0.997) | D (0.007) | D (0.94) | T (0.025) | D (0.683) | D (0.958) | D (1.028) | D (0.743) | |
| L444P | D (-5.92) | D (0.707) | T (0.005) | D (3.19) | D (1) | T (0.412) | D (-5) | D (0.002) | D (0.992) | D (3.16) | D (1) | D (7.949) | T (9.681) | D (24.8) | D (0.996) | D (0.059) | D (0.918) | T (0.999) | NA | D (0.974) | D (1.11) | D (0.858) | |
| L444R | D (-5.91) | D (0.707) | T (0.005) | D (3.19) | D (1) | D (0.986) | D (-4.7) | D (0.001) | D (0.983) | D (3.16) | D (1) | D (7.949) | T (9.681) | D (24.6) | D (0.997) | D (0.224) | D (0.962) | T (0.999) | D (0.795) | D (0.981) | D (1.078) | D (0.891) | |
| L66Q | D (-5.7) | D (0.732) | T (0.003) | T (1.735) | D (1) | T (0.031) | T (0.14) | T (0.532) | T (0.38) | D (3.32) | D (1) | D (2.768) | T (8.21) | T (11.94) | T (0.915) | T (-0.414) | D (0.815) | D (1) | D (0.475) | D (0.785) | D (0.297) | D (0.503) | |
| N188K | D (-5.78) | D (0.706) | D (0) | D (2.545) | D (1) | T (0.079) | D (-4.39) | D (0.006) | D (0.529) | T (-2.24) | T (0.966) | T (0.056) | T (8.044) | T (18.28) | T (0.79) | T (-0.965) | D (0.729) | T (0.721) | D (0.776) | D (0.905) | D (0.552) | D (0.587) | |
| N188S | D (-5.71) | D (0.706) | D (0) | T (1.56) | D (1) | T (0.019) | D (-3.05) | T (0.241) | T (0.366) | T (1.25) | D (1) | D (3.36) | T (7.407) | T (0.013) | T (0.46) | T (-0.71) | D (0.816) | T (0.859) | D (0.241) | D (0.843) | D (0.425) | D (0.494) | |
| N192K | D (-5.49) | D (0.706) | T (0.01) | T (1.705) | D (0.783) | T (0.055) | T (-0.59) | T (1) | T (0.411) | D (2.5) | T (0.996) | T (0.542) | T (5.875) | T (4.408) | T (0.74) | T (-0.544) | D (0.53) | T (0.886) | D (0.334) | D (0.73) | D (0.468) | D (0.464) | |
| N382K | D (-6.07) | D (0.707) | D (0) | D (3.96) | D (1) | D (0.999) | D (-5.56) | D (0.002) | T (0.483) | D (3.33) | D (1) | D (2.007) | T (8.413) | D (27.7) | D (0.998) | D (0.449) | D (0.961) | T (0.618) | D (0.949) | D (0.983) | D (1.087) | D (0.916) | |
| P122L | D (-6.45) | D (0.706) | D (0) | D (3.75) | D (1) | D (1) | D (-9.27) | D (0) | D (0.914) | D (3.55) | D (1) | D (8.802) | T (10.764) | D (25.6) | D (0.998) | D (0.575) | D (0.98) | T (0.999) | D (0.881) | D (0.988) | D (1.016) | D (0.962) | |
| Q497R | D (-3.64) | D (0.732) | T (0.053) | T (0.895) | D (0.631) | T (0.005) | T (-0.92) | T (0.203) | D (0.666) | D (3.14) | D (1) | T (0.524) | T (9.639) | T (16.37) | T (0.911) | T (-0.423) | D (0.85) | T (0.947) | D (0.192) | T (0.476) | T (-0.258) | D (0.508) | |
| R120W | D (-7.03) | D (0.706) | D (0) | D (3.67) | D (1) | D (0.874) | D (-6.84) | D (0) | D (0.911) | D (3.55) | D (1) | D (4.353) | T (10.764) | D (28.6) | D (0.998) | D (0.412) | D (0.966) | T (0.282) | D (0.807) | D (0.993) | D (0.979) | D (0.922) | |
| R163Q | D (-5.57) | D (0.706) | T (0.004) | T (0.835) | D (0.967) | T (0.025) | T (-1.56) | T (0.312) | T (0.054) | T (1.53) | T (0.167) | T (0.555) | T (4.202) | D (20.4) | D (0.991) | T (-0.496) | T (0.475) | T (0.003) | D (0.215) | D (0.839) | D (0.512) | T (0.352) | |
| R285H | D (-6.27) | D (0.707) | D (0) | D (3.8) | D (1) | D (0.998) | D (-4.79) | D (0.001) | D (0.929) | D (3.68) | T (0.995) | D (6.847) | T (11.025) | D (32) | D (0.999) | D (0.539) | D (0.971) | D (1) | D (0.903) | D (0.987) | D (1.03) | D (0.942) | |
| S(-24)G | D (-5.61) | T (0.543) | T (0.248) | T (-0.695) | T (1) | T (0) | T (0.19) | T (1) | T (0.052) | T (0.256) | T (0) | T (0.066) | T (6.318) | T (0.001) | T (0.48) | T (-1.653) | T (0.001) | D (1) | D (0.041) | D (0.574) | D (0.07) | T (0.252) | |
| S107L | D (-5.69) | D (0.732) | T (0.006) | D (2.025) | D (0.787) | D (0.96) | D (-4.68) | D (0.003) | D (0.679) | D (3.25) | D (1) | D (5.033) | T (10.105) | D (29.8) | D (0.998) | D (0.329) | D (0.921) | D (1) | D (0.858) | D (0.95) | D (0.978) | D (0.755) | |
| S196P | D (-5.52) | D (0.706) | D (0.001) | D (2.435) | D (0.997) | T (0.021) | T (-2.23) | D (0.031) | D (0.872) | T (-3.28) | T (0.095) | T (0.158) | T (8.27) | T (11.51) | T (0.957) | T (-0.914) | T (0.165) | T (0.822) | D (0.237) | D (0.85) | D (0.179) | D (0.618) | |
| S271G | D (-6.13) | D (0.706) | D (0) | D (3.325) | D (0.98) | T (0.316) | T (-1.77) | D (0.042) | D (0.853) | D (3.51) | D (1) | D (5.31) | T (8.547) | T (14.81) | T (0.974) | D (0.137) | D (0.883) | T (0.406) | D (0.801) | D (0.958) | D (1.13) | D (0.737) | |
| V191G | D (-5.8) | D (0.706) | T (0.006) | T (1.215) | D (1) | T (0.001) | D (-3.81) | D (0.01) | D (0.915) | D (2.5) | T (0.995) | D (4.227) | T (7.355) | T (0.206) | T (0.639) | T (-0.747) | D (0.913) | T (0.985) | D (0.422) | D (0.816) | D (0.508) | D (0.624) | |
| V460M | D (-5.71) | D (0.706) | D (0) | D (2.04) | D (0.999) | D (0.701) | T (-1.81) | D (0.046) | D (0.899) | D (3.16) | T (0.936) | T (1.94) | D (12.16) | D (27.8) | D (0.996) | D (0.143) | D (0.868) | T (0.979) | D (0.79) | D (0.957) | D (1.056) | D (0.818) | |
| W393C | D (-6.42) | D (0.707) | D (0) | D (3.48) | D (1) | D (0.996) | D (-10.57) | D (0.001) | D (0.95) | D (4.95) | D (1) | D (7.172) | D (16.05) | D (35) | D (0.993) | D (0.836) | D (0.996) | D (1) | D (0.791) | D (0.99) | D (0.997) | D (0.949) | |

*Note:* Allele name according to the traditional nomenclature associated with Gaucher disease, referring to the processed protein with the first 39 amino acids removed. Data are presented as a prediction (score). A total of 22 computational methods were used to predict the pathogenicity of non-synonymous *GBA* variants, including nine functional prediction methods, four conservative methods, and nine ensemble methods. Since the MutationTaster score represents the accuracy of the pathogenicity prediction results, we could directly differentiate deleterious and tolerable nonsynonymous variants. All scores other than MutationTaster scores were converted to binary functional effect predictions of “deleterious” and “tolerable" based on the pathogenicity threshold for each tool.

Abbreviations: D: deleterious; T: tolerable.

**Supplementary Table 2.** *GBA* variants identified in *GBA*-PD group

| **Cases** | **Allele name**  **(HGVS)** | **Allele name**  **(traditional)** | **Amino acid**  **change** | **Nucleotide**  **change** | **Exon** | **Class of**  **variant** | **RS (dbSNP)** |
| --- | --- | --- | --- | --- | --- | --- | --- |
| 1 | D438H | D399H | p.Asp438His | c.1312G>C | 10 | mild | - |
| 1 | G416C | G377C | p.Gly416Cys | c.1246G>T | 10 | mild | - |
| 1 | V499M | V460M | p.Val499Met | c.1495G>A | 11 | mild | rs369068553 |
| 1 | F252I | F213I | p.Phe252Ile | c.754T>A | 7 | severe | rs381737 |
| 1 | G364R | G325R | p.Gly364Arg | c.1090G>A | 9 | severe | rs121908305 |
| 1 | L422Pfs*3 | L383Pfs*3 | p.Leu422Profs*3 | c.1265_1319del | 10 | severe | rs80356768 |
| 5 | L483P | L444P | p.Leu483Pro | c.1448T>C | 11 | severe | rs421016 |
| 1 | L483R | L444R | p.Leu483Arg | c.1448T>G | 11 | severe | rs421016 |
| 1 | N421K | N382K | p.Asn421Lys | c.1263C>A | 10 | severe | - |
| 1 | P161L | P122L | p.Pro161Leu | c.482C>T | 6 | severe | rs79637617 |
| 3 | R159W | R120W | p.Arg159Trp | c.475C>T | 6 | severe | rs439898 |
| 1 | R324H | R285H | p.Arg324His | c.971G>A | 8 | severe | rs79696831 |
| 1 | S146L | S107L | p.Ser146Leu | c.437C>T | 5 | severe | rs758447515 |
| 1 | E365K | E326K | p.Glu365Lys | c.1093G>A | 9 | risk | rs2230288 |
| 1 | D419N | D380N | p.Asp419Asn | c.1255G>A | 10 | unknown | - |
| 2 | L224R | L185R | p.Leu224Arg | c.671T>G | 7 | unknown | - |
| 1 | L363V | L324V | p.Leu363Val | c.1087C>G | 9 | unknown | rs1272814464 |
| 1 | L105Q | L66Q | p.Leu105Gln | c.314T>A | 5 | unknown | - |
| 1 | N231K | N192K | p.Asn231Lys | c.693T>A | 7 | unknown | - |
| 1 | P14P | P(-26)P | p.Pro14Pro | c.42T>G | 3 | unknown | rs1392613829 |
| 1 | Q536R | Q497R | p.Gln536Arg | c.1607A>G | 12 | unknown | rs750779755 |
| 5 | R202Q | R163Q | p.Arg202Gln | c.605G>A | 7 | unknown | rs398123531 |
| 1 | S52S | S13S | p.Ser52Ser | c.156G>A | 4 | unknown | rs756264143 |
| 1 | S212S | S173S | p.Ser212Ser | c.636A>G | 7 | unknown | rs556008401 |
| 2 | S310G | S271G | p.Ser310Gly | c.928A>G | 8 | unknown | rs1057942 |
| 1 | S403S | S364S | p.Ser403Ser | c.1209C>T | 9 | unknown | rs773947710 |
| 1 | V415V | V376V | p.Val415Val | c.1245C>T | 10 | unknown | rs755952419 |
| 1 | W432C | W393C | p.Trp432Cys | c.1296G>T | 10 | unknown | - |
| 1 | G241R | G202R | p.Gly241Arg | c.721G>A | 7 | complex | rs409652+ |
|  | S235P | S196P | p.Ser235Pro | c.703T>C |  |  | rs1064644+ |
|  | V230G | V191G | p.Val230Gly | c.689T>G |  |  | rs381427+ |
|  | N227K | N188K | p.Asn227Lys | c.681T>G |  |  | rs381418+ |
|  | N227S | N188S | p.Asn227Ser | c.680A>G |  |  | rs364897 |
| 1 | S16G | S(-24)G | p.Ser16Gly | c.46A>G | 3 | complex | rs1141804+ |
|  | L15S | L(-25)S | p.Leu15Ser | c.44T>C |  |  | rs1141802 |
| 1 | R202Q | R163Q | p.Arg202Gln | c.605G>A | 7-11 | complex | rs398123531+ |
|  | L483P | L444P | p.Leu483Pro | c.1448T>C |  |  | rs421016 |

*Note:* Allele name (HGVS) according to the reference sequence NM_001005741 and the Human Genome Variation Society nomenclature (HGVS; http://varnomen.hgvs.org/). Allele name (traditional) according to the traditional nomenclature associated with Gaucher disease, referring to the processed protein with the first 39 amino acids removed.

Abbreviations: *GBA*-PD, *GBA*-related PD; PD, Parkinson disease; ACMG: American College of Medical Genetics and Genomics.

**
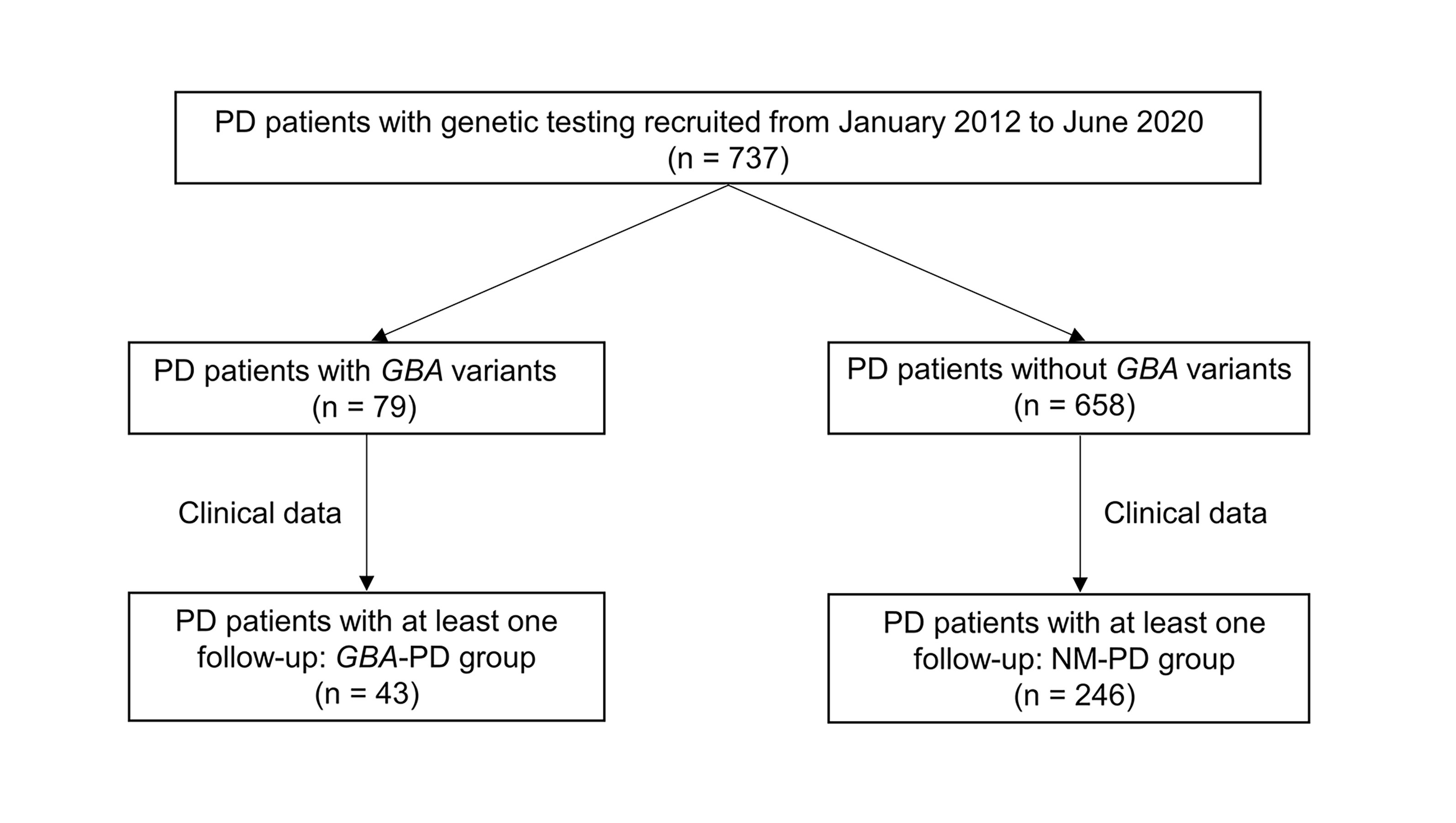
**

**Supplementary Figure 1.** The flow chart of participant screening. Abbreviations: *GBA*-PD, *GBA*-related PD; NM-PD, non-*GBA*-mutated PD; PD, Parkinson disease.

**
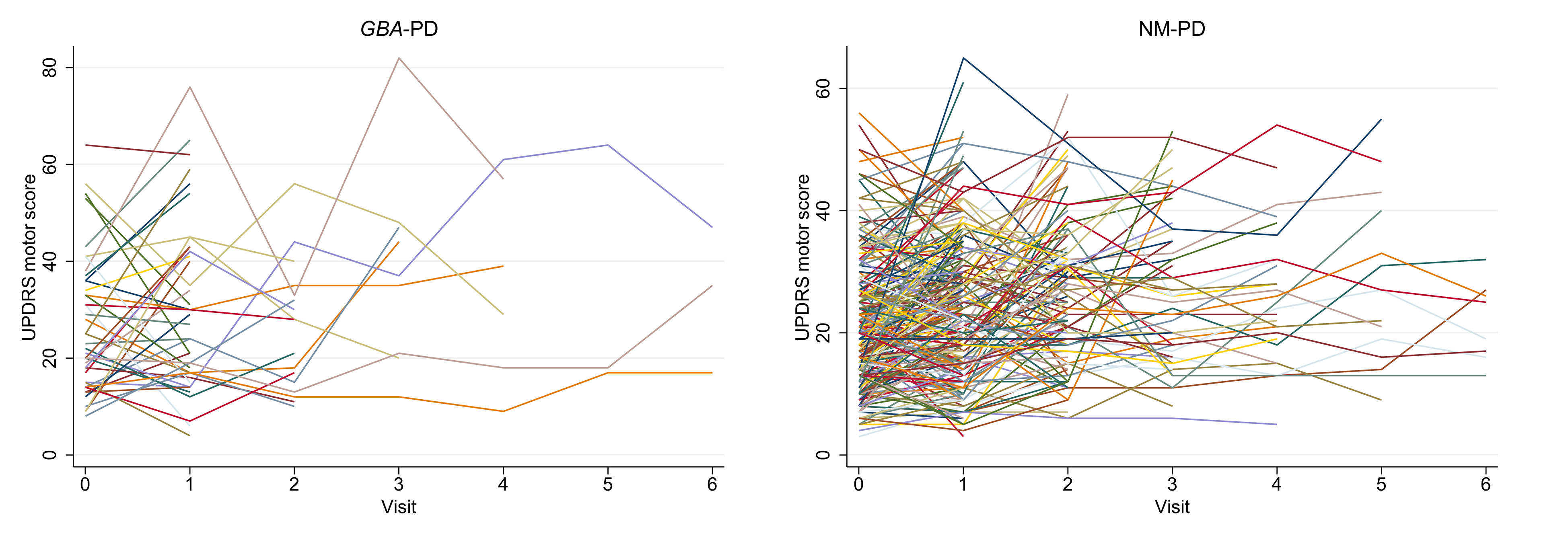
**

**Supplementary Figure 2.** Spaghetti plots of changes in UPDRS motor score across visits. Abbreviations: *GBA*-PD, *GBA*-related PD; NM-PD, non-*GBA*-mutated PD; PD, Parkinson disease; UPDRS, Unified Parkinson's Disease Rating Scale.
